# Chemistry of the Metals—Copper

**Published:** 1855-07

**Authors:** R. N. Wright


AMERICAN JOURNAL

OF

### DENTAL SCIENCE.

Vol. V. NEW SERIES—JULY, 1855. No. 3.

### ORIGINAL COMMUNICATIONS.

### ARTICLE I.

*Chemistry of the Metals—Copper.* By Professor R. N. Wright, A. M., M. D.

(Continued from page 178.)

We present to our readers another of the old metals, viz. Copper, but one which within, comparatively, a short time, has become exceedingly interesting in many respects to the lovers of science.

Its name seems to have been derived from an island in the Mediterranean, (Cyprus,) in the vicinity of the Levant; hence, *Cuprum*, the title by which it is known to scientific men. By some it is said to have been *first discovered* in the island of Cyprus, and by others, to have derived its name from the fact that it was *first manufactured there*, by the Greeks. It must have been to the ancients, what *wrought* iron is to the present age; for, they seem to have used it very extensively in the manufacture of implements of husbandry and weapons of warfare, as well as culinary utensils.

Copper is obtained in a great variety of forms; it is found

*native* or uncombined; united with sulphur in the form of sulphuret and sometimes with *sulphuric acid*, as sulphate; with hydro-chloric acid, carbonic acid, arsenic and phosphoric acids, or hydrochlorates, carbonate, arseniate and phosphate of copper, the most common ones, perhaps, being the oxyds, sulphurets and the carbonates.

Without entering further into the natural history of the metal, we will proceed to mention some of the leading characteristics of the cupreous compounds most commonly met with, as well as the form of crystallization assumed under ordinary circumstances. In Tomlinson, I find the following: "Copper is sometimes found in the metallic state, but more frequently in combination with the metalloids, with oxygen, sulphur and arsenic. The sulphurets are most abundant, and from them the commercial demands are almost exclusively supplied. The sulphuret, combined with the sulphuret of iron, forms the well known *yellow copper* ore. Native copper often crystallizes under the form of small regular octohedrons; these may be also obtained when copper is slowly precipitated from solutions by voltaic electricity. Copper affects the same crystalline form, when a considerable mass, melted in a crucible, is left to cool slowly; the part which remains liquid, after reposing some time, having been poured off. Chemically pure copper may be obtained by passing a stream of hydrogen gas over the oxyd heated to redness in a glass tube. If a small quantity of the oxyd is operated on in this way, the reduced metal is obtained in thin films, which, by reflected light, present the characteristic red colors of copper, but by transmitted light are of a beautiful green. Copper is very malleable and ductile; it may be beaten out into thin leaves, or drawn out into fine wire; it has great tenacity, being in this respect only inferior to iron. Its density varies from 8.78 for cast copper, to 8.96 for rolled or hammered copper. It has a peculiar taste, and by friction, evolves a disagreeable odor. It melts at a strong red heat, which has been fixed, by Daniell, at 1996, Fahrenheit. At a white heat, it passes off in vapors, which burn in the air with a green flame. At a white heat, copper decomposes the vapor of water. At ordinary temperatures, this metal does not oxydize in dry air, but changes quickly in moist air ; it then becomes covered with a strongly adherent green crust."

When copper is highly heated and plunged into cold water, or rapidly cooled in a current of air, desquamation occurs, the result being an imperfectly formed oxyd; but when the heated metal is immersed in water, there is no decomposition of that liquid. When a small quantity of sulphuric acid is added to good nitric acid, and copper dissolved in the mixture, on diluting it, and immersing a clean piece of iron, we can procure perfectly pure copper, which needs only washing and fusion.

*Reduction of Copper Ores.*—We extract the contents of the above named paragraph from the distinguished chemist of Great Britain, Brande, he says, "The following is an outline of the process by which the ores of copper are reduced, as carried on upon a very large scale, near Swansea, where the chief part of the Cornish ores are brought to the state of metal. The ore, having been picked and broken, is heated in a reverberatory furnace, by which arsenic and sulphur are driven off. It is then transferred to a smaller reverberatory, where it is fused, and the slag which separates, being occasionally removed, is cast into oblong masses, which are used as a substitute for bricks. The impure metal collected at the bottom of the furnace is granulated by letting it run into water; it is afterwards remelted and granulated two or three times successively, in order further to separate impurities, which are chiefly sulphur, iron and arsenic, and ultimately cast into oblong pieces, called pigs, which are broken up, roasted and melted with a portion of charcoal, in a refining furnace. Malleability is here conferred upon the copper, and its texture improved by stirring the metal with a pole of green wood, generally birch, which causes great ebulition and agitation; assays are occasionally taken out, and the metal originally crystalline and granular, when cold, now becomes fine and close, so as to assume a silky polish when the assays are half cut through and broken. The metal is now cast into cakes about 12 inches wide by 18 inches long. The whole process of refining the copper and toughening it, by *poling*, requires much care and attention; and if it be over poled, the metal is even rendered more brittle than in its original state. The effect of poling has not been satisfactorily explained, it may consist in the separation of a small portion of oxyd of copper, and the effect of over poling, may possibly depend upon the combination of the copper with a portion of the carbon. Copper for brass-making is granulated by pouring the metal through a perforated ladle into water; when this is warm, the copper assumes a rounded form, and is called *bean shot;* but if a constant supply of cold water is kept up, it becomes ragged, and is called *feathered shot.* Another form into which copper is cast, chiefly for exports into the East Indies, is in pieces of the length of six inches and weighing about six ounces each: the copper is dropped from the moulds immediately on its becoming solid, into a cistern of cold water, and thus by a slight oxydation of the metal, the sticks acquire a rich red color on the surface. This is called *Japan copper.* A large quantity of copper is rolled into sheets and sheathings, both for export and home consumption.*"

Next in order, we propose to introduce the *Alloys of Copper*, some of which are of great importance in the arts; and here we mean to make liberal extracts from the excellent work of Brande, reminding our readers of the motive which prompted the production in the Journal of this series of articles on the "Chemistry of the Metals," viz. the placing within reach of all who may be interested, a series of papers compiled with the greatest care, not only from observation, but every other reliable source, and in every instance where quotations are made, (of any extent at least,) allowing the *author* to speak for himself.

*Alloys of Copper.*†—Many of these compounds are of great use in the arts, especially those with zinc and tin, and with silver and gold. The alloys of copper with potassium and sodium, have not been particularly examined. Davy formed them, and ascertained that they decomposed water. Manganese was com-

* For an account of the English method of carrying on the reduction of copper, the curious are referred to Mr. Vivian's papers, Ann. of Philadelphia, N. S., vol. 113.

† Brande, Eng. Ed., pp. 826, 827, 828 and 829.

bined with copper by Bergman, and the properties of the alloy were examined by Gmelin, (Göttingen Commentaries, 1787.) Iron and copper combine with difficulty: 100 parts of gray cast iron and 5 of copper yield a very hard alloy, of which Rinmann has proposed to make anvils. With zinc and tin, copper forms brass and bronze. With cadmium, the alloy is brittle and harsh; it is decomposed by keeping it in fusion, when the cadmium volatilizes. The alloy of copper and cobalt has not been examined, With nickel, copper yields *pakfong* and *German silver.* Dumas gives the following view of the composition of different pakfongs.

| | Spoons & Forks. | Knife handles and Snuffers. | for Lamination. | Articles requiring soldering. | White, but harsh and hard. | Chinese Pakfong. |
|---|---|---|---|---|---|---|
| Copper, | 50 | 55 | 60 | 57 | 53 | 40.4 |
| Nickel, | 25 | 22 | 20 | 20 | 22 | 31.6 |
| Zinc, | 25 | 23 | 20 | 20 | 23 | 25.4 |
| Lead, | 0 | 0 | 0 | 3 | 0 | 0.0 |
| Iron, | 0 | 0 | 0 | 0 | 2 | 2.6 |
| | 100 | 100 | 100 | 100 | 100 | 100.0 |

German nickel is used by the manufacturers of packfong; it is broken up and mixed with the granulated copper and zinc, taking care that copper forms the upper and lower stratum in the crucible: the whole is covered with charcoal powders, and fused in a wind furnace; it is stirred and kept for sometime in fusion, at the risk of evaporating part of the zinc. When the clippings and filings are remelted, 3 or 4 per cent. of zinc are added to compensate for volatilization."

"*Brass.*—In making this important alloy, the metals are ususally united by mixing granulated copper with calamine and charcoal; the mixture is exposed to heat sufficient to reduce the calamine and melt the alloy, which is then cast into plates. The relative proportions of the two metals, vary in the different kinds of brass, and some contain, too, a little lead and tin. The composition of the principal varieties is shown in the following table:

| | For Turning. | | For Gilding. | | For Wire. | For Hammering. | For Fine Casting. | |
|---|---|---|---|---|---|---|---|---|
| Copper, | 61.6 | 65.8 | 63.7 | 82 | 66.5 | 70 | 91.2 | 91.7 |
| Zinc, | 35.3 | 31.8 | 33.6 | 18 | 33.1 | 30 | 5.6 | 5.0 |
| Lead, | 2.9 | 2.2 | 2.5 | 1 | 0.4 | 0 | 1.8 | 2.3 |
| Tin, | 0.2 | 0.2 | 0.2 | 3 | 0.0 | 0 | 1.4 | 1.0 |
| | 100.0 | 100.0 | 100.0 | 104 | 100.0 | 100 | 100.0 | 100.0 |

Brass is very malleable and ductile when cold, and its color and little liability to rust, recommend it in preference to copper for many purposes of the arts; its specific gravity varies from 7.9 to 8.9, and exceeds the mean of its components, as shown by the following results, (Dumas.)

| | Copper. | Zinc. | Actual Density. | Calculated Density. |
|---|---|---|---|---|
| I. | 70 | 30 | 8.443 | 8.390 |
| II. | 80 | 20 | 8.940 | 8.560 |

According to Regnault, the specific heat of a brass composed of 71 copper, 27.6 zinc, and 1.3 lead, and affording traces of tin, is 0.09391, (Ann. de Ch. et Ph. lxxiii, 33.) According to Sage, a beautiful brass may be made by mixing 50 parts of oxyd of copper, 100 of calamine, 400 of black flux, and 30 of charcoal powder; melt them in a crucible until the blue flame is no longer seen around the cover, and when cold, a button of brass is found at the bottom, of a golden color, and weighing one-sixth more than the pure copper, obtained from the above quantity of oxyd. The presence of iron and of tin, in brass, should be carefully avoided, when they may interfere with its ductility or malleability.

*Tutenag*, is said to be an alloy of copper, zinc, and a little iron, and *tombac*, *dutch gold*, *similos*, Prince Rupert's metal, and pinch-beck, are alloys, containing more copper than exists in brass, and consequently made by fusing various proportions of copper with brass. According to Wiegleb, Manheim gold consists of 3 parts of copper and 1 of zinc. A little tin is sometimes added, which, though it may improve the color, impairs the malleability of the alloy. An alloy of 576 parts of copper, 59 of tin, and 48 of brass, is equal to brass in hardness, and may be worked with the same facility; it was used by Mr. Bate, for the new standard measures, as being less liable than brass to oxydizement." (Phil. Trans. 1826.)

* *Bronze.*—This alloy is much harder than copper, and was employed by the ancients to make swords, hatchets, &c., before the method of working iron was generally understood. The art of casting bronze statues, may be traced to the most remote antiquity, but it was first brought to a certain degree of refinement by Theodorus and Roecus, of Samos, about 700 years before the christian era, to whom the invention of modelling is ascribed by Pliny. The ancients were well aware that by alloying copper with tin, a more fusible metal was obtained, that the process of casting was thereby rendered easier, and that the statue was harder and more durable, and yet they frequently made them of copper nearly pure, because they possessed no means of determining the proportions of their alloys, and because by their mode of managing the fire, the copper became refined in the course of melting, as has happened to many founders in our own days. It was during the reign of Alexander, that bronze statuary received its greatest extension, when the celebrated artist, Lysippus, succeeded by new processes of moulding and melting, to multiply groups of statues to such a degree, that Pliny called them the *mob of Alexander.* Soon afterwards, enormous bronze collossuses were made, to the height of towers, of which the isle of Rhodes, possessed no less than one hundred. The Roman consul, Mutiamus, found 3000 bronze statues at Athens, 3000 at Rhodes, as many at Olympia, and at Delphi, although a great number had been previously carried off from the last town."

"In forming such statues, the alloys should be capable of flowing readily into all parts of the mould, however minute; it should be hard, in order to resist accidental blows—be proof against the influence of the weather, and be of such a nature as to acquire that greenish oxydized coat upon the surface, which is so much admired upon the antique bronzes, called *patina antiqua.* The chemical composition of the bronze alloy, is, there-

* De Ure, pp. 280, vol. 1, Am. Ed.

fore, a matter of the first moment. The brothers Keller, celebrated founders in the time of Louis XIV, whose *chefs d'œuvre*, are well known, directed their attention towards this point, to which too little importance is attached at the present day. The statue of Desaix, in the place Dauphine, and the column in the place Vendome, are noted specimens of most defective workmanship, from mismanagement of the alloys of which they are composed. On analyzing separately, specimens taken from the bass-reliefs, on the pedestal of the column, from the shaft, and from the capital, it was found that the first contained only 6 per cent. of alloy, and 94 of copper, the second much less, and the third only 0.21. It was, therefore, obvious, that the founder, unskillful in the melting of bronze, had gone on progressively refining his alloy, by the oxydizement of the tin, until he had exhausted the copper, and that he had then worked up the refuse scoriæ in the top of the column; the cannons, which the government furnished him for casting the monument, consisted of

| | | |
|---|---|---|
| Copper, . . . . . . . . . | 89 | 360 |
| Tin, . . . . . . . . . | 10 | 040 |
| Lead, . . . . . . . . . | 0 | 102 |
| Silver, zinc, iron and loss, . . . . | 0 | 498 |
| | 100 | 000 |

The moulding of several of the bass-reliefs, was so ill executed, that the chiselers employed to repair the faults, removed no less than 70 tons of bronze, which was given them, besides 300,000 francs for their work. The statues made by the Kellers, at Versailles, were found, on chemical analysis, to consist of

| | No. 1. | No. 2. | No. 3. | The mean. |
|---|---|---|---|---|
| Copper, | 91.30 | 91.68 | 91.22 | 91.40 |
| Tin, | 1.00 | 2.32 | 1.78 | 1.70 |
| Zinc, | 6.09 | 4.93 | 5.57 | 5.53 |
| Lead, | 1.61 | 1.07 | 1.43 | 1.37 |
| | 100.00 | 100.00 | 100.00 | 100.00 |

The analysis of the bronze of the statue of Louis XV, was as follows:

| | | |
|---|---|---|
| Copper, . | 82.45. | Its specific gravity was 8.482 |
| Zinc, . . | 10.30 | |
| Tin, . . | 4.10 | |
| Lead, . . | 3.15 | |
| | 100.00 | |

The alloy most proper for bronze medals, which are to be afterwards struck, is composed of from 8 to 12 parts of tin, and from 92 to 88 of copper, to which if 2 or 3 parts in the 100 of zinc be added, they will make it assume a finer bronze tint. The alloy of the Kellers is famous for this effect. The medal should be subjected to three or four successive stamps of the press, and be softened between each blow by being heated and plunged into cold water. The bronze of bells or bell metal, is composed, in a hundred parts, of copper, 78; tin, 22. This alloy has a fine compact grain, is very fusible and sonorous. The other metals sometimes added, are rather prejudicial, and merely increase the profit of the founders. Some of the English bells, consist of 80 copper, 10.1 tin, 5.6 zinc, 4.3 lead; the latter metal, when in such large quantities, is apt to cause isolated drops, hurtful to the uniformity of the alloy.

The chinese gong, *(tshoung,)* according to the analysis of Klaproth, contains nothing but copper and tin, in the proportions of 78 of copper, 22 of tin, specific gravity 8.815. This alloy when newly cast, is as brittle as glass, but by being plunged at a cherry-red heat into cold water, and confined between two discs of iron, to keep it in shape, it becomes tough and malleable. Cymbals, consist of copper, 80 parts; tin, 20.

Bronze vessels naturally brittle, may be made tenacious by the same ingenious process, for which the world is indebted to Mr. Darcet. Bronze mortars, for pounding, have their lips tempered in the same way. Ancient warlike weapons of bronze were variously compounded; swords were formed of 87½ copper, 12½ tin, in 100 parts; the springs of the balistæ consisted of 97 copper, and 3 tin."

I find in the new edition of the work, from which the above copious and interesting facts have been extracted, (in Ure's dictionary,) a receipt for imparting the antique bronze color to figures made of these alloys—the receipt is as follows:

| | |
|---|---|
| Sal ammoniac, . . . . . | 2 drachms. |
| Salt of Sorel, (bin-oxalate of potash,) . | ½ " |
| Colorless vinegar, . . . . . | 14 ounces. |

A hair pencil being dipped into this solution and pressed gently between the fingers, the article to be colored must be gently warmed, and coated with the solution, a sufficient number of times to impart the desired shade of color.

In mixing the metals for making the above alloys, some precautions are to be carefully observed. Those metals which require the greatest amount of heat for their fusion, should be heated first, and then the others added in the order of their degree of fusibility, and again, they should be kept on the fire no longer than is requisite for their perfect incorporation. Another important part of the process, and perhaps that requiring the most skill, is to effect the refrigeration of the mass after it has been run into the moulds, as speedily as possible, for if they are not cooled rapidly, there is danger of the metals separating according to the order of their densities, which of course, would materially injure the alloy.

To be continued in the next number, when we propose to give in full, the process for the deposition of copper, by galvanism, to be followed by a description of the natural and artificial compounds of the metal.

